# Research on collaborative edge network service migration strategy based on crowd clustering

**DOI:** 10.1038/s41598-024-58048-0

**Published:** 2024-03-26

**Authors:** Junjie Cao, Zhiyong Yu, Bin Xue

**Affiliations:** 1 Xi’an Research Institute of High Technology, Xi’an, 710025 People’s Republic of China; 2 College of Information and Communication, National University of Defense Technology, Wuhan, 430035 People’s Republic of China

**Keywords:** Service migration strategy, Crowd intelligent devices, Markov decision process, Deep reinforcement learning, Computational science, Computer science, Information technology

## Abstract

The innovative application of Crowd Intelligent Devices (CIDS) in edge networks has garnered attention due to the rapid development of artificial intelligence and computer technology. This application offers users more reliable and low-latency computing services through computation offloading technology. However, the dynamic nature of network terminals and the limited coverage of edge servers pose challenges, such as data loss and service interruption. Furthermore, the high-speed mobility of intelligent terminals in the dynamic edge network environment further complicates the design of computation offloading and service migration strategies. To address these challenges, this paper explores the computation offloading model of cluster intelligence collaboration in a heterogeneous network environment. This model involves multiple intelligences collaborating to provide computation offloading services for terminals. To accommodate various roles, a switching strategy of split-cluster group collaboration is introduced, assigning the cluster head, the alternate cluster head, and the ordinary user are assigned to a group with different functions. Additionally, the paper formulates the optimal offloading strategy for group smart terminals as a Markov decision process, taking into account factors such as user mobility, service delay, service accuracy, and migration cost. To implement this strategy, the paper utilizes the deep reinforcement learning-based CCSMS algorithm. Simulation results demonstrate that the proposed edge network service migration strategy, rooted in groupwise cluster collaboration, effectively mitigates interruption delay and enhances service migration efficiency.

## Introduction

The rapid development of wireless communication technology and the popularity of the Internet of Things (IoT) have led to an exponential growth in the number of smart terminals. These terminals are based on multi-intelligence, sensing and monitoring devices, sensors, and embedded systems, forming a cluster of smart terminals. The large-scale IoT user community has become a contributor of data and services in cyberspace, leading to the emergence of a new form of IoT-based group intelligent collaboration^1^. IoT data access has greatly improved, resulting in explosive growth and forming multi-source, massive, multi-dimensional, and heterogeneous IoT big data. The application scenarios of IoT have become increasingly diverse, including computationally intensive and latency-sensitive applications such as smart manufacturing, smart logistics, virtual reality, augmented reality, and earthquake relief. Thanks to the mobile edge computing paradigm, computation resources are shifted to locations close to terminals or data sources to meet real-time data processing, security privacy, and big data requirements, which greatly improves the efficiency of IoT applications while reducing network congestion and bandwidth pressure^2,3^.

However, as more intelligent terminal groups are become accessible, the mobile uncertainty characteristic of these groups and the limited coverage of edge servers can necessitate switching to a new server area before the source server completes its computation task. This can easily lead to service disruption or even more serious consequences, such as service migration caused by user mobility. The contradiction between the limited coverage of a single edge server and the mobility of multiple smart terminals can lead to network performance degradation, which in turn significantly impacts the quality of service or even interrupts ongoing edge services. This is the first challenge faced by edge networks^4^. In multi-source heterogeneous networks, edge servers are deployed in the edge network, and their performance is closely related to the high-speed mobility of smart terminals. Due to the user's mobility and the limited coverage of edge servers, communication between them may need to go through multiple hops, which can seriously impact the quality of service (QoS), especially for demanding services^5^. To minimize the negative impact of this QoS degradation, services must be dynamically migrated to a better location, preferably closer to the new user's location. Service migration techniques are becoming increasingly complex, making arithmetic offloading, balancing, and network QoS closely related to service migration. This is the second challenge faced^6^. In dynamic and open real-world environments, traditional siloed decision-making architectures are insufficient for handling complex tasks. Collaborative decision-making organizations are required to systematically improve the effectiveness of group-wise systems. These organizations are used to cope with network congestion and migration failures caused by group maneuvers, posing a challenge for the crowd intelligence collaborative model and application. Therefore, achieving seamless service migration is crucial to ensure service continuity when CIDS is moving. This presents another important challenge for edge computing in the next step.

This paper suggests a strategy for edge service migration in high-speed mobile scenarios, using group-intelligence sub-cluster collaboration. The strategy takes into account the impact of CIDS high-speed mobile state characteristics on service migration and leverages group-intelligence advantages to enable collaborative work between intelligences. Resource scheduling is achieved through controllers, and a prediction model based on Markov decision-making is adopted to improve the efficiency and accuracy of service migration.

The main contributions of this paper are as follows:

1. The model architecture for collaborative service migration in high-speed mobile environments is proposed for the first time. In such environments, the availability and performance of services may rapidly decline. Therefore, this strategy dynamically adjusts the position of services based on the speed and direction of the agents, better meeting their needs. By optimizing the location and allocation of services, we ensure the best quality and improve the overall system performance. Through the collaboration and learning of multiple agents, we can better predict their behavior, enhancing the efficiency and accuracy of service migration;
2. A collaborative management mechanism for clustering is proposed, consisting of cluster heads, backup cluster heads, and regular users. Various scenarios that the group may encounter are identified, and corresponding decision strategies and processes are designed. When the environment changes, this strategy can update the migration plan of services in real-time, ensuring that the services always meet the needs of the agents. This dynamic adjustment capability enables the strategy to adapt to various complex and uncertain real-world scenarios.
3. We propose an algorithm based on deep reinforcement learning for the CCSMS algorithm. This algorithm transforms the optimization problem into a partially observable Markov decision process framework and utilizes deep reinforcement learning methodology to design a service migration strategy for groupwise cluster collaboration. This strategy automates the process of adjusting and optimizing service migration, thereby reducing the likelihood of manual interventions and errors.

The remaining parts of the paper are organized as follows. Part II provides a review of research progress on service migration strategy both domestically and internationally. Part III introduces the system model and provides a detailed description and problem statement of the proposed strategy mechanism. Part IV presents the innovative design of the algorithm. Part V describes the simulation scenario, experimental setup, and evaluates the performance of the proposed method. Finally, Part VI summarizes the work done in the entire paper.

## Related work

### Crowd intelligence

Crowd intelligence is an intelligent approach to solving complex problems through collective intelligence and collaboration. It is inspired by the intelligent behavior of groups of social organisms in nature. These organisms demonstrate a holistic intelligent behavior by dividing labor, coordinating mutually, and evolving synergistically to successfully complete various complex tasks. The self-organization, self-adaptation, and self-learning ability of crowd intelligence have brought great inspiration to scholars at home and abroad. They have used mathematical and computer tools to simulate crowd intelligence behaviors and explore the intrinsic mechanisms and models of crowd intelligence emergence and evolution from different perspectives. Wang et al.^7^ proposed an observer-based control strategy that solves the control consistency problem of the coding-decoding communication protocol (CDCP) of networked multi-intelligent body systems in discrete time. This strategy offers consistency control of distributed systems and provides an effective solution. Zhao et al.^8^ proposed a distributed optimization method based on multi-intelligent body reinforcement learning. This method helps improve the optimization efficiency and performance of distributed systems by maximizing the long-term overall utility of the network while guaranteeing the quality of service of heterogeneous cellular network devices. Xu et al.^9^ designed an event-triggered and privacy-based protection algorithm to guarantee the security consistency of multi-intelligent body systems under denial of service (DoS) attacks. This algorithm is crucial for the security and stability of distributed systems. The GP algorithm evaluates the fitness value of an individual using short-term reinforcement learning. It efficiently searches the feature space and improves algorithm efficiency by representing a set of feature sets with intelligent individuals of a population. The fitness value of each individual is evaluated based on the average performance of short-term reinforcement learning. Tian et al.^10^ conducted an in-depth study on the controllability and observability of multi-intelligent body systems with heterogeneous and switching topologies. They considered the controllability of the system from a graph-theoretic point of view and stated that a system is controllable if the concatenated graphs of all possible topologies are controllable^11^. This research provides important theoretical support and practical guidance for analyzing the controllability and observability of distributed systems.

The aforementioned research indicates that as the Internet advances, collaboration becomes increasingly intricate in terms of scenes, components, and constraints. Collaborative computing has gradually integrated artificial intelligence technologies such as big data and statistical machine learning, and the theory and model based on crowd intelligence collaboration are being improved to handle more complex real-world problems. While research on crowd intelligence collaboration holds important theoretical and practical value, there is a lack of research on service migration environments. Therefore, it is imperative to conduct research on crowd intelligence collaboration service migration to further advance the field and promote the practical application of crowd intelligence technology, which holds significant importance^21^.

### Service migrations

Considering the high-speed mobility of smart devices, the optimization of service migration and resource scheduling in edge networks is becoming increasingly complex. Ensuring system latency and service continuity is crucial in this context. Fortunately, there have been previous research efforts to improve the success rate of service migration, with lightweight container virtualization techniques gaining significant attention. Karhula et al.^12^ demonstrated the migration of IoT edge functions using Docker and CRIU for checkpointing. However, their proposed approach has a limitation of unsynchronized persistent files between the source (ES(src)) and target (ES(tgt)) edge servers, and it does not account for state changes after the checkpoint is established. Nadgowda et al.^13^ and others proposed a Docker-based stateful migration. The proposed approach involves transferring the storage state to the target edge server (ES(tgt)). To minimize service downtime during local filesystem migration, data is transferred using dual-band transfer, suggesting the use of a network filesystem. However, if network-attached storage is unavailable, the migrated service may experience frequent errors in the background-replicated files, leading to a degradation in the quality of service (QoS). Al-dhuraibi et al.^14^ proposed an automated vertical scaling system for Docker containers called Elastic Docker. Although it differs from containerized service migrations, Elastic Docker performs hot migrations when there are no remaining resources on the host for scaling. In dynamic migration in Elastic Docker, file system differences are transferred first, followed by the transfer of memory state. Yu et al.^15^ proposed a container migration approach with logging and replay. This approach logs the changes during image transfer and replays them on the target edge server (ES(tgt)), while stopping the source edge server (ES(src)) and resuming the target edge server (ES(tgt)). Although this migration scheme migrates persistent files, it does not migrate certain memory pages, which may be a limitation for stateful applications.

Literature^16,17^ achieved a trade-off between the number of signaling and transmission delay by predicting mobility and reduced transmission delay by service migration, but the performance of these algorithms relies heavily on the mobility trajectories obtained from the predictions. In order to alleviate the dependence on the mobility trajectory, several works that do not need to be based on the mobility trajectory have been proposed, e.g., in the literature^18,19^, which assume that the user's mobility obeys a Markovian process, model the service migration problem as a Markovian decision-making problem, and propose an optimal threshold policy to minimize the migration cost of the system. Although these methods are detached from the reliance on movement trajectories, they are performed in the context that the global information of the default system is known. However, in real MEC scenarios, global information is difficult to obtain, and even if it is obtained, it requires a significant cost. In order to move away from the dependence on global information, literature^20^ proposes a migration decision algorithm based on Markov process to minimize the migration cost under the condition of guaranteeing user QoS. In addition, literature^21^ uses MAB theory to learn the information on the user side and the base station side, and then based on the information obtained from the learning, Liaplov optimization method is used to make the migration decision under energy consumption limitation in order to minimize the average user delay.

In summary, although compute offloading and service migration face many major challenges, a number of researchers have already studied these challenges. However, in most of the existing studies on offloading decisions and resource allocation, the scenarios are based on multi-user, single compute node, which is quite different from real application scenarios. Another problem is that some of the studies do not fully consider the characteristics of the services to be migrated and the migration techniques used lack comprehensive consideration of server nodes and user mobility, and are not applicable to high mobility scenarios. In order to address these shortcomings and migrate containerized services efficiently, different migration techniques should be selected based on the characteristics and requirements of the services. Whenever a service migration decision needs to be made, the trade-offs between the potential benefits and costs of migration need to be considered simultaneously. In order to increase the success rate of service migration and get better benefits, several studies have adopted edge computing resource scheduling approaches based on predictive mobility patterns^22^, such as using Markov Decision Processes (MDPS) and different optimization techniques, as well as prediction or monitoring relying on state information. Markov Decision Processes are one of the most commonly used trade-off models, where a decision maker takes possible actions when given a state, each of which is rewarded or rewarded accordingly, and so the whole process involves constant consideration of what action to take in order to maximize the reward. However, all existing prediction-based approaches have difficulty in meeting the quality of service requirements of group IWTs. On the one hand, such methods tend to consider only the behavior prediction of single intelligences, while ignoring the interaction and collaboration between intelligences. In real-life scenarios, the interactions between intelligences often have an important impact on the overall performance of the system. For example, in an ITS, the movement pattern of a single vehicle may be affected by other vehicles, while in a smart city, the movement pattern of a crowd may be affected by a variety of factors such as infrastructure. Therefore, we need an approach to consider the interactions and collaborations between intelligences in order to predict the movement patterns more accurately; on the other hand, existing approaches tend to focus only on the efficiency of service migration while ignoring the quality of services. In many application scenarios, such as autonomous driving and telemedicine, the quality of service is crucial^23^. Therefore, we need an approach to guarantee the service quality during and after the service migration process. Finally, existing approaches may have efficiency problems when dealing with large-scale intelligences and dynamic environments. In real systems, millions or even billions of intelligences may need to be monitored and predicted in real time, which requires efficient and real-time algorithms to handle.

## System modelling and problem description

### System modelling

**Figure 1:**
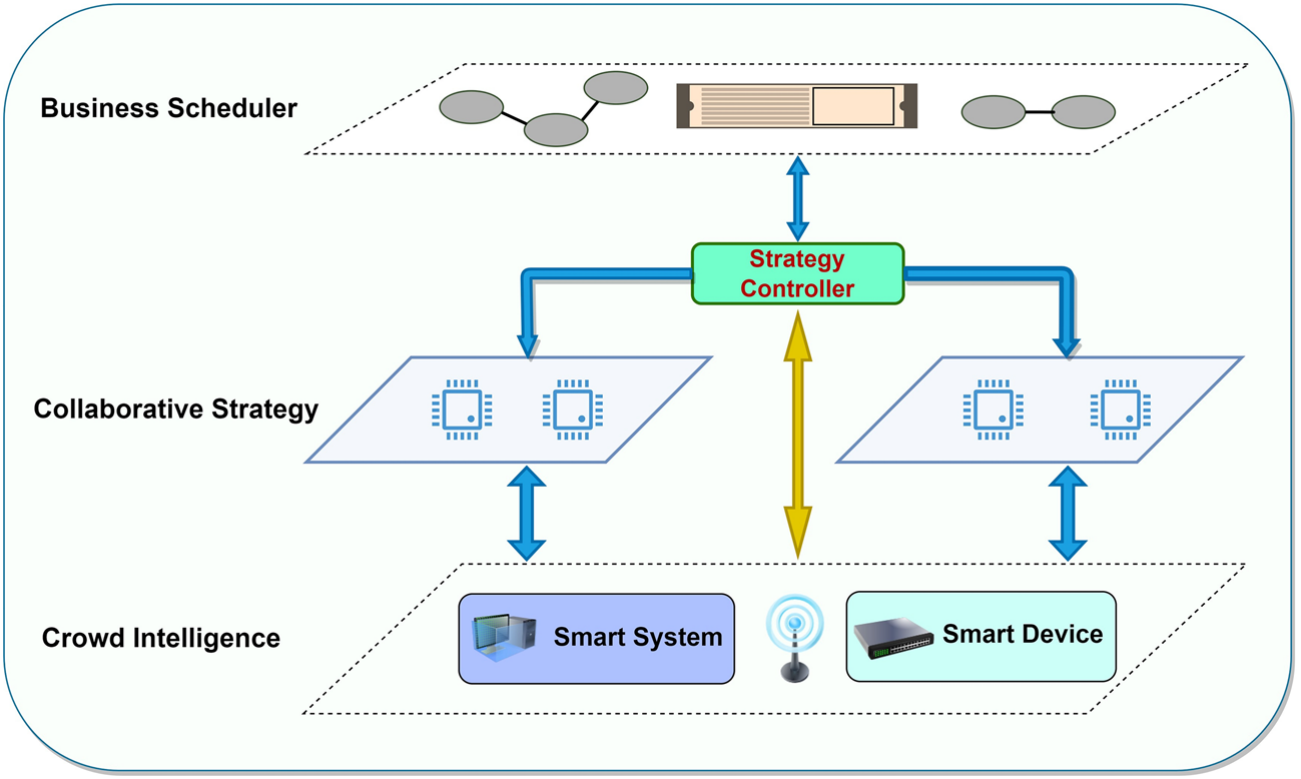
Cluster Collaboration Architecture for Crowd Intelligent Devices.

In order to solve the above problems, we abstract and analyze the service migration work of multi-intelligence in this section. The management architecture of cluster-intelligence sub-cluster collaborative service migration is shown in Fig. 1. Based on the concept of multi-layer collaboration, the mobility management and service orchestration contents are fully integrated in the distributed environment for effective tracking, management and optimization of high-speed mobile crowd intelligent devices or users. Keeping a close eye on the goal of service migration, various resources, tasks and processes are choreographed and scheduled to provide support for intelligent decision-making, further improve the efficiency and effectiveness of service migration, and achieve service continuity and efficiency. The model is divided into three distinct layers. The crowd intelligence layer, situated at the bottom, encompasses the edge network's crowd intelligence devices; the middle layer is the collaboration strategy layer, which is responsible for the decision-making and instruction issuance of the whole service migration strategy; and the top layer is the service orchestration layer, which is responsible for the overall scheduling, allocation and management of resources. We assume that each base station is equipped with one edge server and all edge servers have identical performance. The base station is responsible for information forwarding and computation tasks (the edge server is responsible for computation).

We work with time slots in the service migration process considering the powerful computing power of edge servers. Further, we analyze the computational requirements of the services to be migrated, allocate a specific time slot for each service, migrate the service to the target node and start it within the specified time slot, and monitor and make necessary adjustments during the service operation. Adopting the time-slot working method can effectively coordinate the migration between multiple services to ensure time synchronization and the services obtain the necessary computing resources^24^.

### Service migration strategy

**Figure 2:**
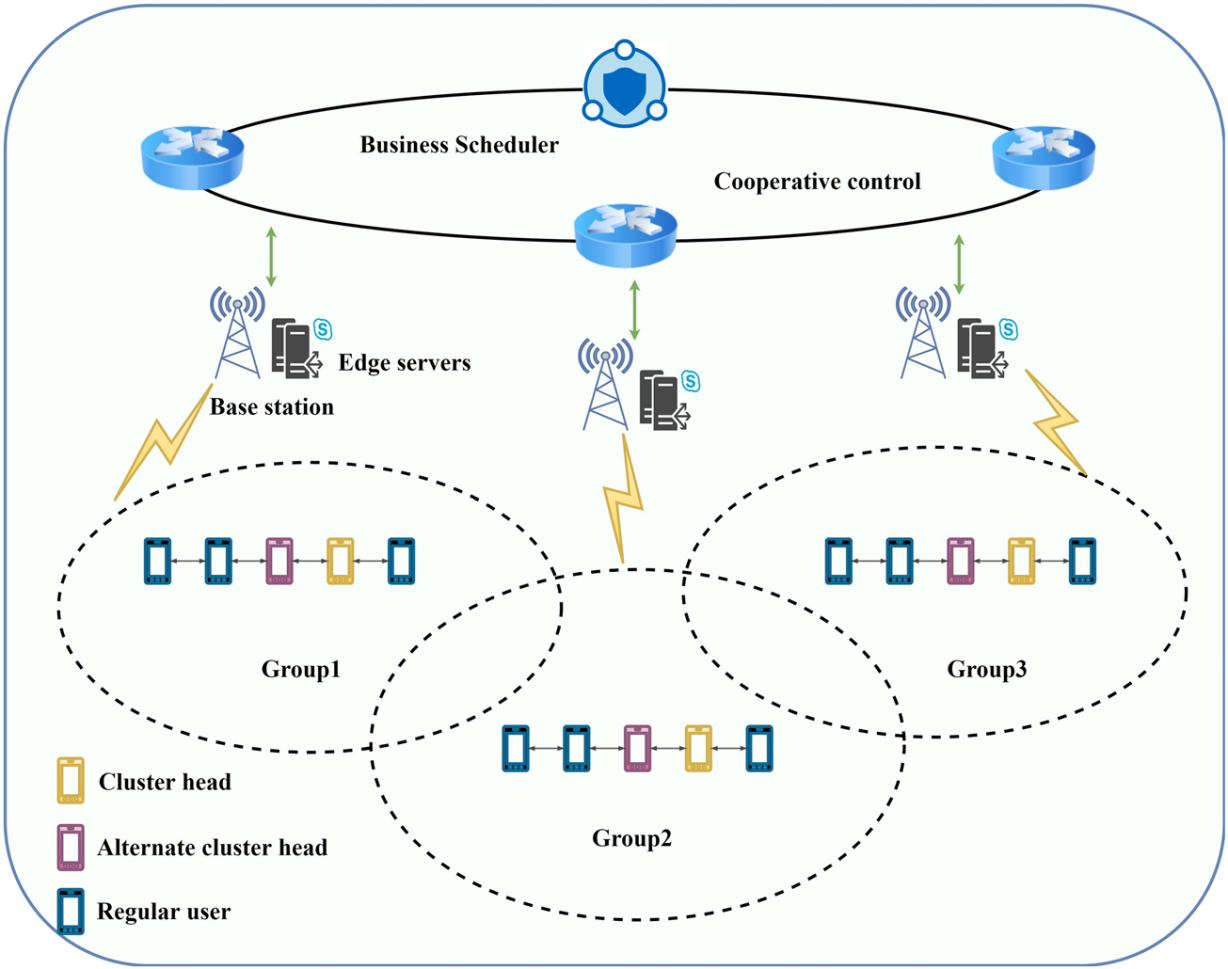
Crowd intelligent device sub-cluster collaborative service migration.

The high-speed movement of CIDS takes place from the coverage of one edge server to another, in which the service preferences and action paths of multiple CIDSs may be extremely similar or phase similar, which brings us new inspirations and makes it possible to effectively integrate the migration tasks and efficiently improve the migration efficiency^25^. Based on the service migration architecture mentioned in the previous section, this chapter proposes a dynamic service migration strategy based on Cluster Intelligence Divided Cluster Collaboration (CIDSDC), where CIDS devices with the same or similar migration tasks are grouped together to form a CIDSDC service migration architecture model, as shown in Fig. 2.

In this model, CIDS can be sliced into three categories, i.e., cluster heads, alternate cluster heads, and ordinary users. The cluster head is responsible for sending status information to other users and collects all other user link quality information and link quality information with the base station, and sends it to the base station after collecting the information, which is used for evaluating the next mobile migration decision. The crowd-smart device with the most neighbors acts as the cluster head in the cluster, and all the rest of the users are defined as normal users; The alternate cluster head periodically sends the status information of the users in the cluster and collects all the other users' link quality information, and unlike the cluster head, the alternate cluster head does not have to collect the link quality information with the base station because the alternate cluster head acts as a backup for the cluster head at any time, and most of the related cluster-based services are handled by the cluster head primary to handle. Once the cluster head is unable to perform its duties, the alternate cluster head needs to take over to perform the functions of the cluster head; Regular users periodically measure the status information from the base station, the cluster head, and the alternate cluster head, and when the measurement is completed, the measurement report is sent to the cluster head and the alternate cluster head, so that group maintenance and further service migration decisions can be made.

The CIDS periodically measures the reference signal from the base station. If a migration event is triggered, the CIDS sends a measurement report to the base station, and the cluster head reports all the collected link quality information together to the base station. The base station receives these measurement reports and reports the results to the cooperative controller, which in turn decides whether to execute the migration mechanism. If it is determined that the cluster should be migrated, a request is sent by the service orchestration layer to the target base station, and if the target base station determines that it can be afforded, it sends a response signal. After the response the base station sends a migration command to the cluster along with information about the target base station. The cluster then performs synchronization and access to the target base station. Finally, the cluster sends a successful migration notification and the migration is complete.

In order to provide highly reliable continuous services in a dynamic network, we construct the network as a time-slot model, which combines service scheduling to allocate resources to different services, using the time-slot model for scheduling and management. At the same time, the strategy introduces a Markov decision process to predict the future state based on the current state of the groupwise devices, and when the state of the node changes, the model immediately enters the beginning of the next time slot, thus selecting the best migration decision^26^. The whole service migration process is described as follows:

1. Exploration phase

Periodically the CIDS detects the signals sent by the surrounding base stations, and if the signal strength and quality received by the CIDS is lower than the threshold or is about to leave the area covered by a particular edge server when they are in the process of high speed movement, a service migration event will be triggered, which marks the beginning of the service migration effort.

As mentioned earlier, the cluster is maintained by periodically measuring the signals from the cluster head, the alternate cluster head, and all the link quality information collected by the cluster head and reported to the base station. The base station receives the measurement reports and determines what strategy to adopt based on the link quality^27^.

Upon triggering a service migration event, the base station receives the measurement report for further judgement, and link quality should be considered at the same time. At the same time, the base station sends the ID information, measurement object, and report configuration status information to the CIDS, the ID information is used to ensure the correct measurement identification of the ordinary user, and the measurement object and report configuration are to ensure the uniformity of the data. However, it is undeniable that there is a possibility of loss of report data in case of conflict, but retransmission leads to unacceptable time delays. Therefore, it is permissible to send blank report information, and when the base station receives a blank report, it can refine the report by estimating the missing data with the help of historical reports, through linear or logarithmic interpolation.

(2) Interaction phase

The CIDS starts scanning to select a suitable target BTS and sends a service request report to the upper level collaborative controller. The collaborative controller collaborates with the edge server to make a judgement on whether to carry out the migration decision or not. If it determines to migrate, the cooperative controller sends a request message to the target server, and the target server replies with a response message if it can accommodate the migration request from the CIDS. After the response, the cooperative controller sends a migration command to the CIDS, which contains information about the acquired target server.

(3) Synergy phase

One of the most critical steps in the entire migration process is collaboration, which requires reasonable matching and grouping of CIDS to form cluster-based collaborative groupings, and real-time monitoring of the status of the equipment and the execution of tasks. Necessary dynamic adjustments and optimizations are made based on the feedback information. At the same time, due to the existence of a large number of mobile terminals migrating requests at similar times in the scenario set in this paper, it is easy for request conflicts and collisions to occur, which will bring about greater latency. In order to solve this problem, we propose a packet-based mobility management mechanism using the packet negotiation mechanism.

**Figure 3:**
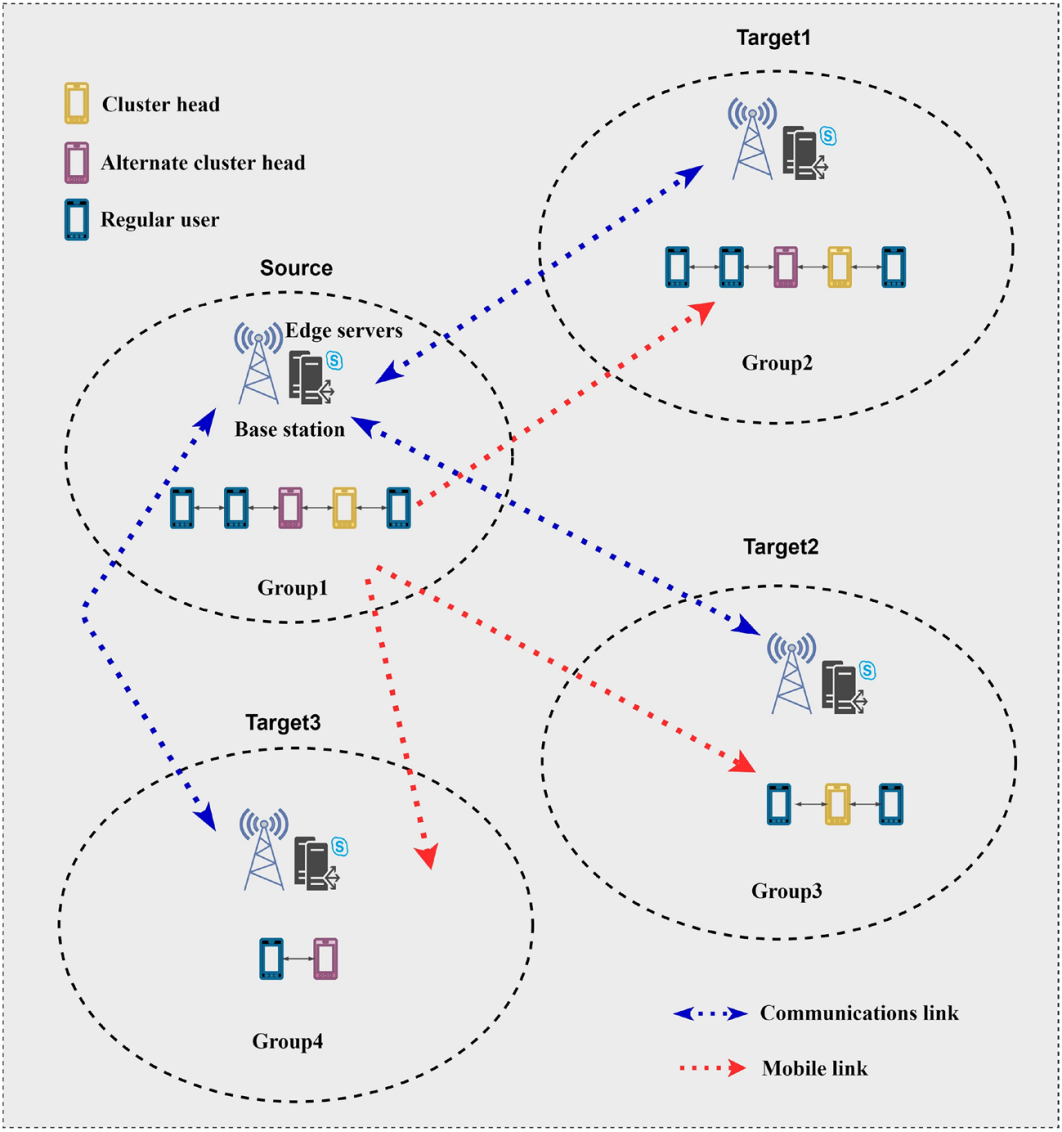
Cluster Collaborative Interaction.

CIDS periodically interact with the BS for information and record these interactions in the measurement report, which is fully considered in the migration decision. If the target server signal quality becomes better than the source server, migration is triggered, and if multiple CIDSs have the same request information, we group these CIDSs reasonably, that is, the CIDSs that satisfy the same triggering event in the same cycle form a cluster. In this case, the services within the cluster are switched from the source base station to the target base station, a process known as full switching. The process of transferring cluster 1 from the source server to the target 1 server is shown in Fig. 3. After the handover is completed, if some of the CIDS triggers a new handover event, the cluster is forced to be decomposed into a number of subgroups, which are then used to perform the corresponding handovers, such as the process of regrouping and migrating cluster 1 from the source server to the target 2 and 3 servers in Fig. 3. Of course, this is the ideal situation, within a triggering event cycle, the CIDS in the group that has priority to complete the handover will remain in the half handover state, at this time, although in the coverage area of the target base station, but still provided by the source base station. At the same time, the half-switching timer is activated and waits for other group members in the group to complete all switching. If the other group members can finish before the end of the timer, the group as a whole will switch to the target BTS together, which is the result we are looking forward to. If some members fail to enter the target base station to complete the handover at the end of the timer, the group is forced to be decomposed into subgroups, and then the handover process is executed sequentially, a process we call group handover^28^. In the following we set up several special cases and discuss them separately:

- Alternate cluster head election mechanism

After sending peer discovery signals in all cluster members and measuring the signals sent by others, the cluster head selects an alternate cluster head after collecting reports from all other users in the group. The mechanism of selection is that the one with the most neighbours is preferred as the alternate cluster head and the alternate cluster head acts as the legal successor of the cluster head. Therefore, periodic re-elections are required to ensure that the alternate cluster head is always appropriate.

(b) Primary and alternate cluster head swapping

The primary and alternate cluster head swapping mechanism is activated to ensure the robustness of the service migration cluster network when the quality of the cluster head links is poor or the quality of the links of most of the members of the cluster is poor. After the swap, the cluster head becomes a alternate cluster head until the next swap mechanism is activated or the alternate cluster head election mechanism is activated. If both the primary and alternate cluster head swaps and the alternate cluster head election mechanism occur at the same time, the policy specifies that the alternate cluster head election takes place first, followed by the primary and alternate cluster head swaps. The reason for this is that the new standby cluster head that is elected is more suitable to lead the cluster. The alternate cluster head has status information about the cluster and is therefore able to perform the duties of a cluster head.

(c) Bad state connection mechanism

In a cluster, if the link status between a regular user and the main and standby cluster head is persistently poor for a given period of time, the base station will activate the bad state connection mechanism, which will kick the user out of the cluster and thus maintain the service migration benefits of the cluster. The user kicked out of the cluster then participates normally in the service migration of the subgroups in other cycles. The link state information between the cluster head, the alternate cluster head, and the regular user may also be in a poor condition, in which case the mechanism of re-dividing the subgroups is activated, and the base station will force the original cluster to be divided into two groups, with the original cluster head and the alternate cluster head acting as the cluster heads of the subgroups, respectively.

(d) Time synchronization

Firstly, it is divided into a series of fixed-length time slots, the length of which can be adjusted according to the demand of tasks, resource availability and system performance requirements; then available network resources are allocated to different time slots through the co-controller and service orchestration management, and the allocation of time slots needs to take into account the resource demand of devices and services, the availability of time slots, and the availability of resources in order to ensure that each device and service has sufficient resources to execute; finally, in each time slot, appropriate computational and storage resources are allocated to ensure the sequence and timeliness of data transmission and processing, as well as to monitor and control the resource usage^29^.

(4) Execution phase

At the end of the collaboration, the CIDS starts data transmission with the target server and informs the base station that it has been successfully completed once the switchover is complete. Once the handover fails, it is necessary to reselect and connect to the base station in order to resume the data transmission. Due to the long duration of reselection and connection, this delay caused by switchover failure is absolutely intolerable. Therefore, how to reduce the service migration failure rate is a critical problem in migration strategies.

### Problem description

This paper presents research in the application scenario of crowd intelligent sensing, where crowd intelligent terminals move at high speed and work together in complex and changing environments to achieve real-time and accurate sensing of the environment. This puts extreme demands on service migration techniques, especially in terms of latency and migration interruptions. The increase in latency may lead to the failure of real-time sensing data. Since crowd intelligent sensing requires processing and analyzing a large amount of data in real time, the migration of edge computing services must reduce latency as much as possible to ensure the timeliness and accuracy of the data. Migration interruptions, on the other hand, may trigger service discontinuity, thus affecting the overall effect of group intelligent perception. During the migration process, measures need to be taken to minimize service interruption time, and avoid service instability or data loss due to migration. By analyzing and predicting services and making preparations for migration of services in advance, and at the same time, using the flexibility of collaborative grouping, the efficiency and robustness of migration can be greatly improved, which greatly solves the application requirements of crowd-smart device sensing. The consideration of limited resources and mobility of smart terminals will inevitably lead to changes in network dynamics thus affecting the service migration strategy and performance. In this section, we first model the service migration problem in a heterogeneous edge network, followed by optimizing the algorithm design for the modelled problem^30^. We assume that there is only one edge server in a region, and the edge migration strategy ultimately determines when to start the migration, the question of which server to migrate to, and how to reduce the total delay of the migration to minimize the total delay and the system overhead.

#### Computation models

We consider a typical heterogeneous network environment under a coverage area which contains a base station BS, an edge server ES, $N=\left\{\mathrm{1,2}\dots n\right\}$ as the set of group smart terminals, and $S=\left\{\mathrm{1,2}\dots s\right\}$ as the set of edge servers. The ES can provide computation and migration services only for the users in the wireless coverage area. Assuming that the network operates in discrete time slots, define the time as the set $T=\left\{\mathrm{0,1},2\dots t\right\}$ and denote the length of each time slot by $\tau$. Groupwise devices with the same or similar migration tasks are grouped to form the set of group clusters, which we denote by $G=\left\{\mathrm{1,2}\dots g\right\}$.

We assume that the task arrivals all obey a Poisson distribution with $\lambda \in \left[\mathrm{0,1}\right]$, and the computation task of each smart terminal cannot be divided into sub-tasks, i.e., the whole computation demand is to be executed locally or at the edge server. We set ${\xi }_{i}\left(t\right)\in \left\{\mathrm{0,1}\right\},i\in N$ to denote the task arrivals in time slot $\tau$. When ${\xi }_{i}\left(t\right)=0$ means no new task, and ${\xi }_{i}\left(t\right)=1$ means a new task has been generated. ${x}_{i}\left(t\right)\in \left\{\mathrm{0,1}\right\}$ denotes the migration strategy of smart terminal n in time slot $\tau$. Let ${x}_{i}\left(t\right)=0$ mean migration is prohibited, and let ${x}_{i}\left(t\right)=1$ mean the initiating the migration strategy. We define the resource set, the bandwidth resource as $\{ F_{0}^{max} ,\,B_{0}^{max} \}$, where $F_{0}^{max}$ denotes the maximum computational capacity and ${B}_{0}^{max}$ denotes the maximum available bandwidth resource. To simplify the complexity of the analysis, we use a ternary to represent the tasks to be reached, denoted as $\left(\mu ,\omega ,\varphi \right)$, where $\mu$ is the size of the task in bits, $\omega$ is the computational density in number of CPU cycles to be executed, and $\varphi$ is the maximal computational latency constraint. For easy reference, some important notations are summarised in Table 1.

**Table 1:** Notations.

Notation	Description	Notation	Description
$N$	Smart terminal collection	$D$	Delay
$S$	Edge server collection	$v$	Travelling speed
$T$	Time collection	${l}_{n}$	Position
$G$	Cluster collection	${P}_{n}^{u}$	Wireless channel transmission power
${x}_{i}\left(t\right)$	Migration strategies	${\sigma }^{2}$	Noise power
${\xi }_{i}\left(t\right)$	Mission arrival status	${\rho }_{n,s}^{mgt}(t)$	Migration cost
${F}_{0}^{max}$	Maximum computing power	$\alpha$	Weighting weight factor
${B}_{0}^{max}$	Maximum available bandwidth	$\beta$	Learning rate
$\mu$	Size of the task	$\varrho$	Neural network parameter matrix
$\omega$	Calculation density	$\vartheta$	Calibration system parameters
$\varphi$	Maximum computation delay constraint	W	Experience pool
${f}_{n}^{l}$	Local computing resources	$\mathcal{H}(\pi (\cdot \left|s\right.)$	Entropy of the strategy
${f}_{n}^{s}$	Server-side computing resources	$L\left(\theta \right)$	Loss function

#### Delay models

- Local computing delay

Assuming that ${f}_{n}^{l}$ denotes the local computing resources of the Intelligent Terminal and ${\mu }_{0}^{local}$ is the amount of locally processed tasks. Therefore, the local computing latency is:

1 $${D}_{n}^{l}=\frac{{\mu }_{0}^{local}}{{f}_{n}^{l}}$$

The total local computation latency of the intelligent groups within the subcluster is:

2 $${D}_{g,n}^{l}={\sum }_{n\in N,g\in G}\frac{{\mu }_{0}^{local}}{{f}_{n}^{l}}$$

(b) Server computation delay

Assuming that ${f}_{n}^{s}$ denotes the server-side computational resources and ${\mu }_{1}^{s}$ is the number of tasks processed by the server, the server computational latency is:

3 $${D}_{n}^{s}=\frac{{\mu }_{1}^{s}}{{f}_{n}^{s}}$$

If the maximum computational delay ${D}_{g,n}^{l}+{D}_{n}^{s}>\varphi$, it will be penalised, denoted as:

4 $${\kappa }_{n,s}\left(t\right)={\sum }_{n\in N,s\in S}1\left\{{D}_{g,n}^{l}+{D}_{n}^{s}>\varphi \left|{x}_{i}\left(t\right)=1\right.\right\}$$

(c) Service migration preparation delay

The CIDS moves at high speed from one point, and when it is about to leave the area covered by a certain edge server, i.e., the signal strength and quality are lower than the threshold, the service migration trigger will be activated. According to the intelligent terminal travelling speed $v$ and position ${l}_{n}$, the trigger time for service switching can be obtained as:

5 $${D}_{n}^{trigger}\triangleq \frac{{l}_{n}^{1}-\left|{l}_{n}^{0} mod {l}_{n}^{1}\right|}{v}$$

where $\left|{l}_{n}^{0} mod {l}_{n}^{1}\right|$ denotes the residue of the position at ${l}_{n}^{0}$ and the server coverage area ${l}_{n}^{1}$, which is the relative position of the intelligence terminal in the coverage area.

(d) Cluster queuing delay

In accordance with the strategy of this paper, the crowd intelligent terminal starts to move at high speed from a certain point in the coverage area of this server, and when it is about to leave the area, a migration event will be triggered. So the crowd intelligent devices with the same or similar migration tasks will be grouped to form a cluster, so the formation process of grouping queue ${\mathcal{M}}_{g}(t)$ is:

6 $$ {\mathcal{M}}_{g} \left( {t + 1} \right) = min\left\{ {max\left\{ {{\mathcal{M}}_{g} \left( t \right) - 1\left\{ {x_{i} \left( t \right) \ge 0,0} \right\} + \xi_{g} \left( t \right)} \right\},{\mathcal{M}}_{g}^{max} } \right\},\,g \in G,\,t \in T $$

where $1\left\{x\right\}$ represents the indicator function, which is equal to 1 when condition x is true, and 0 otherwise. ${\mathcal{M}}_{g}^{max}$ denotes the maximum grouping queue limit, i.e., the maximum upper limit. ${\xi }_{g}\left(t\right)={\xi }_{i}\left(t\right)+{\sum }_{g\in G}{\xi }_{i}\left(t\right)$, which denotes the sum of the tasks within the cluster grouping. When the grouping queue reaches the upper limit, it will be forcibly discarded for regrouping. Let ${D}_{g,0}^{s}$ be the average forwarding time of the packet, then the total delay caused by the queuing of cluster subgroup members is:

7 $${D}_{g,n}^{s}={\sum }_{n\in N,g\in G}{D}_{g,0}^{s}$$

(e) Wireless transmission delay

The wireless uplink transmission rate ${r}_{n}^{up}$ is:

8 $${r}_{n}^{up}={B}_{0}^{max}{log}_{2}\left(1+\frac{{P}_{n}^{u}}{{\sigma }^{2}}\right)$$

where ${P}_{n}^{u}$ denotes the wireless channel transmission power and ${\sigma }^{2}$ denotes the noise power, whereby the uplink delay is:

9 $${D}_{n}^{up}=\frac{\mu }{{r}_{n}^{up}}$$

#### Migration cost overhead

Service migration incurs additional cost overheads, which need to be analyzed and calculated by taking into account multiple factors such as task volume, data volume, model complexity, hardware resources and time cost. In this paper, in order to focus on the main factor of task volume, a simplified calculation of migration cost is done. Therefore, the migration cost overhead ${\rho }_{n,s}^{mgt}(t)$ can be expressed as:

10 $${\rho }_{n,s}^{mgt}(t)=\left\{\begin{array}{l}0, \quad \quad \quad { x}_{i}\left(t\right)=0\\ \Upsilon\left|\mu \right|, \quad \, { x}_{i}\left(t\right)=1\end{array}\right.$$

where ${x}_{i}\left(t\right)=0$ mean migration is prohibited, ${x}_{i}\left(t\right)=1$ mean the initiating the migration strategy, $\left|\mu \right|$ is the task size and $\Upsilon$ is a positive coefficient.

From the above analysis, we can get the total system delay as:

11 $${D}_{total}(t)=\sum_{n\in N,s\in S}{D}_{g,n}^{l}+{D}_{n}^{s}+{D}_{n}^{trigger}+{D}_{g,n}^{s}+{D}_{n}^{up}$$

Thus, the system's overhead on the total can be expressed as the sum of the total delay and cost overhead:

12 $${Q}_{total}=\sum_{n\in N,s\in S}{\alpha D}_{total}(t)+{(1-\alpha )\rho }_{n,s}^{mgt}(t)$$

where $\alpha$ is the weighting weight factor.

The proposed cooperative service migration strategy problem based on crowd intelligence is modelled in this paper, which has the optimization objective of minimizing the total delay and cost overhead of the crowd intelligence system, with the aim of improving the average delay performance and overhead cost in the whole system. The proposed optimization problem aims to find the optimal solution of the problem^31^. Therefore, the minimization problem constructed in this paper is described as follows:

13 $$\mathrm{min }\sum_{n\in N,s\in S}{Q}_{total}$$

$$s.t. { \xi }_{i}\left(t\right)\in \left\{\mathrm{0,1}\right\}$$

$$ {x}_{i}\left(t\right)\in \left\{\mathrm{0,1}\right\}$$

$$ \alpha \in (\mathrm{0,1})$$

$$ \alpha \ge 0$$

$$\Upsilon \ge 0$$

$${\kappa }_{n,s}\left(t\right)\in (\mathrm{0,1})$$

$$ {\sum }_{n\in N}^{s\in S} F\cdot 1\left\{{x}_{i}\left(t\right)\ge \mathrm{0,0}\right\}{\le F}_{0}^{max}$$

$$ {\sum }_{n\in N}^{s\in S} B\cdot 1\left\{{x}_{i}\left(t\right)\ge \mathrm{0,0}\right\}{\le B}_{0}^{max}$$

where, in sequence, the constraints impose limits on task arrival, constraints on whether or not to migrate, constraints on the range of weight factor sizes, requirements for a reasonable range of coefficients in the migration cost overheads, as well as explicit limits on the range of the penalty function, and finally requirements for a reasonable range of computational power and bandwidth. Since ${x}_{i}\left(t\right)$, ${\xi }_{i}\left(t\right)$ are discrete binary values and $F,B$ are continuous values, the optimisation problem is clearly a challenging NP-hard problem.

## Algorithm design

On the basis of the analysis of the high-speed movement characteristics of the crowd-smart system, the goal of this paper is to minimize the system latency and reduce the system cost at the same time. The above problem is difficult to be solved by traditional methods, because it is a typical Markov decision-making process considering that the service migration policy is only related to its current state and not to the past state. In this section, we first introduce the knowledge related to reinforcement learning, then describe the proposed Markov-based decision process, and finally, propose an improved Q-learning service migration decision algorithm based on it.

### Reinforcement learning (RL)

Reinforcement learning, as an important method of machine learning, is different from other learning methods in that, in conducting the training process, the intelligent body needs the corresponding feedback from the environment space, and requires the intelligent body to be able to interact with the environment space, and then optimally make decision-making aids to achieve the goal and maximize the benefit as the action goal. Usually consists of intelligent body, environment space, state space, action space, reward function several parts^32^.

### Deep reinforcement learning (DRL)

As the focus of the classical algorithm Q-learning in reinforcement learning, the goal of Q-learning is to find the optimal strategy to achieve the maximum cumulative reward. By constructing a Q-table to contain state space and action space, the rewards of each action are constantly updated thus updating the Q-table as follows:

14 $$Q\left({S}_{t},{A}_{t}\right)\leftarrow Q\left({S}_{t},{A}_{t}\right)+\beta ({r}_{t}+\gamma {max}_{{A}_{t+1}}Q\left({S}_{t+1},{A}_{t+1}\right)-Q\left({S}_{t},{A}_{t}\right))$$

where $Q\left({S}_{t},{A}_{t}\right)$ denotes the expected value obtained by choosing the action space under the state space $\beta \in (\mathrm{0,1})$ denotes the learning rate, which is used for system convergence control. $\gamma \in \left(\mathrm{0,1}\right)$ denotes as the discount rate, which represents the effect of future rewards on the current rewards. With the change of state space and action space, the Q-table will become more and more huge, and it will become less and less easy to find the Q-table. So the combination of Q-learning and deep neural network (DNN) becomes a new trend, the most representative one is DQN. Thus it has higher sample efficiency than other DRL algorithms with the same strategy, especially in this multi-intelligentsia environment. Assuming that there is a group action of intelligences, a single intelligence cannot know the current operation of other intelligences, and all the accumulated experience is used to train a single DQN, increasing the amount of training data in the environment space. Thereby, each intelligence can reuse past experience values and other intelligences' experience for continuous training, and can also be easily scaled to plan multitasking actions, which is ideally suited for optimizing service migration and route planning at the same time. The DQN has an experience replay function, which allows it to incorporate previous historical training information and learn by updating a small set of samples in the experience pool W when it is necessary to update the deep neural network. The DQN generates a target Q-network which is updated at a slower rate than the current one, making the whole model training more stable^33^. It can be represented as follows:

15 $${y}^{DQN}\left(t\right)={R}_{t}+\gamma {max}_{{A}_{t+1}}{Q}^{\mathrm{^{\prime}}}\left({S}_{t+1},{A}_{t+1};{\theta }^{-}\right)$$

### MDP model

Markov Decision Process MDP is the ideal mathematical model for Reinforcement Learning. Individuals making decisions are called intelligent bodies (Agent), and outside the intelligent body is called the environment (Environment). Intelligent body running in the environment in the environment constantly interact, in the process of interaction, the intelligent body to choose action (action) and execution, the environment to make the corresponding response, change the whole environment and the intelligent body's state (state). The environment also generates corresponding rewards to the intelligent body. All the intelligent body has to do is to choose its action as much as possible to maximize the total reward^34^. Therefore, we use it to model the proposed problem. The key elements of MDP are shown below:

1. System state: the system state at the moment t includes the state information of the edge servers and intelligent terminals in the network:
  16 $${S}_{n,s}(t)=\left\{{S}_{t}^{F B}(t),{S}_{t}^{L}(t),{S}_{t}^{\mu \varphi }(t),{S}_{t}^{{\mathcal{M}}_{g}}(t),{S}_{t}^{{P}_{n}^{u}}(t)\right\}$$
  where ${S}_{t}^{F B}\left(t\right)$ denotes the available resources; ${S}_{t}^{L}(t)$ denotes the location information; ${S}_{t}^{\mu \varphi }(t)$ denotes the task volume and maximum delay constraints; ${S}_{t}^{{\mathcal{M}}_{g}}(t)$ denotes the cluster grouping queue; and ${S}_{t}^{{P}_{n}^{u}}(t)$ denotes the wireless channel transmission power.
2. Policy: Policy is a function of the likelihood of action and guides the choice of decisions. The effectiveness of the policy directly affects the choice of action and thus the overall effectiveness of the system. In this work, we try to obtain the optimal policy through deep reinforcement learning algorithms.
3. Action space: in the system, each intelligence must decide to which edge server coverage area it will move, so the composite action at moment t is:
  17 $${a}_{n,s}\left(t\right)=\left\{{a}_{n,s}^{mgt}\left(t\right),{x}_{i}\left(t\right)\right\}$$
  where ${a}_{n,s}^{mgt}\left(t\right)$ denotes the migration from the source server to the destination server.
4. Reward: when an intelligent body performs an action, it receives feedback from the environment, i.e., a reward. We set the reward function in this paper as the sum of total delay and cost overhead:
  18 $${r}_{t}={D}_{total}\left(t\right)+{\rho }_{n,s}^{mgt}\left(t\right)$$

### Description of the improved algorithm (crowd-smart collaborative services migration strategy, CCSMS)

Although Q-Learning has been widely used, it still has many shortcomings. In the early stage of learning, the maximum operator will bring bias to the Q-value, at the same time, the size of the state space of the problem proposed in this paper is large, and it is impractical if all the action space is stored in the Q-table^35^, so this paper view to improve it from the following aspects:

We try to introduce the idea of maximum entropy reinforcement learning introduced as follows:

19 $${\pi }_{MaxEnt}^{*}=arg{max}_{\pi }\sum_{t}{E}_{\left({S}_{t}{,A}_{t}\right)\sim }{\rho }^{\pi \left[r\left({S}_{t}{,A}_{t}\right)+\alpha \mathcal{H}(\pi (\cdot \left|S\right.)\right]}$$

where $\mathcal{H}(\pi (\cdot \left|s\right.)$ denotes the entropy of the strategy. The parameter a controls the relative importance of rewards and entropy: when it becomes 0, we revert to normal RL. Inspired by this idea we can incorporate a new learning method for Q-Learning, Soft Q-Learning. its algorithm can be formulated as:

20 $${Q}_{soft}\left({S}_{t},{A}_{t}\right)\leftarrow {r}_{t}+\Upsilon {E}_{{S}_{t+1}}\left[{V}_{soft}\left({S}_{t+1}\right)\right] \forall {S}_{t},{A}_{t}$$

21 $${V}_{soft}\left({S}_{t},{A}_{t}\right)\leftarrow \alpha log{\int }_{A}{\text{exp}}\left(\frac{1}{\alpha }{Q}_{soft}\left({S}_{t},{A}^{\mathrm{^{\prime}}}\right)\right)d{A}^{\mathrm{^{\prime}}}.$$

By using this method, convergence to ${Q}_{soft}^{*}$ and ${V}_{soft}^{*}$ can be achieved respectively. When α → 0, Q-learning with a hard max operator can be recovered. For this reason, this is known as Soft Q- learning.

Once we have learnt the Q-functions above, we can obtain the optimal maximum entropy policy:

22 $${\pi }_{MaxEnt}^{*}\left({A}_{t}\left|{S}_{t}\right.\right)={\text{exp}}\left(\frac{1}{\alpha }{Q}_{\mathit{soft}}^{*}\left({S}_{t},{A}_{t}\right)-{V}_{\mathit{soft}}^{*}\left({S}_{t}\right)\right)\propto {Q}_{soft}^{*}\left({S}_{t},{A}_{t}\right)$$

Sampling in ambient space directly using this method is exceptionally difficult, and by employing the Stein Variational Gradient Descent method [Stein Variational Gradient Descent (SVGD)], it is possible to estimate this optimal policy by minimizing the following KL scatter:

23 $${D}_{KL}=\left(\pi (\cdot \left|S\right.)\right)\parallel exp\left(\frac{1}{\alpha }{Q}_{soft}^{*}\left({S}_{t},{A}_{t}\right)-{V}_{soft}^{*}\left({S}_{t}\right)\right)$$

The relative overgeneralization problem is well solved by the maximum entropy strategy, and at the same time there is another problem that has to be solved, that is, in the use of deep neural networks to solve the server migration problem in the case of high-speed movement, used to represent the Q-value, which can be outputted to any state-action correspondences (Q-values) to be stored in the correspondence table, which can effectively improve the feasibility of the problem in the group motion state-space environment. We can make the following approximation:

The relative overgeneralization problem is well solved by the maximum entropy strategy. At the same time there is another problem that has to be solved, which is in the use of deep neural networks to solve the server migration problem in the case of high-speed movement. Used to represent the Q-value, any state-action correspondence (Q-value) can be output to be stored in the correspondence table, which can effectively improve the feasibility of the problem in the group motion state space environment. We can make the following approximation:

24 $${\widetilde{Q}}_{soft}^{*}\left({S}_{t},{A}_{t},\varrho \right)\approx {Q}_{soft}\left({S}_{t},{A}_{t}\right)$$

where $\varrho$ denotes the parameter matrix of the neural network. When the parameters are optimised, the approximated $\widetilde{Q}$ will be close to the true Q value.

The history information of the interaction between the intelligent body and the spatial state of the environment is stored as a tuple in the reply buffer W. In turn, $\varpi$ records are sampled from W to compute the target Q-value in the following way:

25 $${y}_{j}(t\mathrm{^{\prime}})={{R}_{j}+\varsigma min}_{A\mathrm{^{\prime}}}{\widetilde{Q}}_{soft}^{*}\left({S}_{t\mathrm{^{\prime}}},{A}_{t\mathrm{^{\prime}}},\varrho \right)$$

where $j$ denotes a record in the set $\varpi$. The probability that each sample is sampled is:

26 $$P\left(j\right)=\frac{{p}_{j}}{\sum ({p}_{j})}$$

To incrementally update the ${\widetilde{Q}}_{soft}^{*}$ network parameters, we constructed the loss function:

27 $$L\left(\theta \right)=\frac{1}{\varpi *{P\left(j\right)}^{-}\vartheta }\sum_{j=1}^{\varpi }{\left({y}_{j}\left({t}^{\mathrm{^{\prime}}}\right)-{\widetilde{Q}}_{soft}^{*}\left({S}_{t},{A}_{t},\varrho \right)\right)}^{2}$$

where $\vartheta$ represents the parameters of the calibration system, which are used to correct some of the deviations, the above equation cleverly uses the neural network back propagation technique to update the parameters $\varrho$ until the network parameters converge.

**Algorithm 1:**
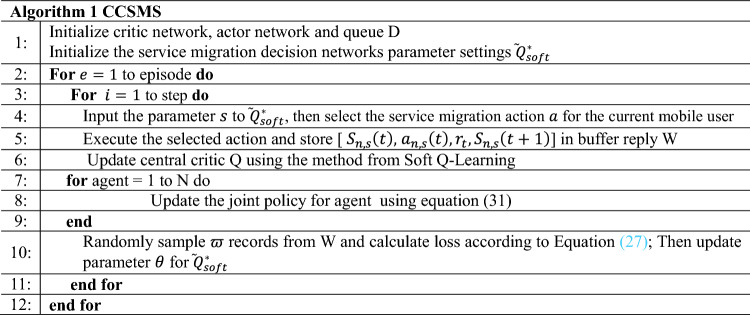
CCSMS

A well-established property of this strategy is that it spreads widely across the action space in continuous control tasks, being able to exploit user mobility information and establish an objective function that minimizes the average task response time. The core part of the improved optimization algorithm in this paper is shown in Algorithm 1.

## Simulation results and evaluation

In order to evaluate the performance of the proposed scheme in this paper, the simulation results are analyzed and discussed in this section.

### Parameter settings and experimental environment

The simulation experiments in this paper are really carried out on a laptop equipped with 3.2Ghz Intel I7-8565 CPU and Python. We have carried out numerical simulation experimental and the data involved in the experiment is shown in Table 2.

**Table 2:** Parameter settings.

Variable	Value range
Network area size	2 km^2^
Number of base stations	1–20
Number of CIDS	1–200
Maximum computing power	2-4GHz
Max bandwidth	1 Gbps
Number of tasks	1–1000
Learning rate	0.0001
${P}_{n}^{u}$	20 w
Size of task	5 kbits
Maximum available task queue	1 ~ 10 task

### Comparison methods

We have chosen three existing more classical and popular deep reinforcement learning algorithms for comparative analysis.

1. DQN algorithm

Deep Q-learning (DQN) is a machine learning method capable of dealing with complex state spaces, which is able to learn from experience and make optimal decisions by combining deep learning and reinforcement learning. In a service migration scenario, DQN can learn the state space of a service and find out the best action to take in various states.

(2) DDQN Algorithm

Distributed Deep Q Learning (DDQN), on the other hand, is an approach that applies deep reinforcement learning to a distributed environment. In a distributed environment, each agent can make decisions independently and also interact with other agents in the environment. In the scenario of service migration, DDQN can be applied to multi-user scenarios, and by uniting the states of all mobile users for training, it can solve the interaction problem in a multi-user environment.

(3) Greedy-SP

It is a traditional greedy algorithm that first finds the current closest path and performs service migration based on resubmission strategy.

### Analysis of results

#### Convergence analysis

**Figure 4:**
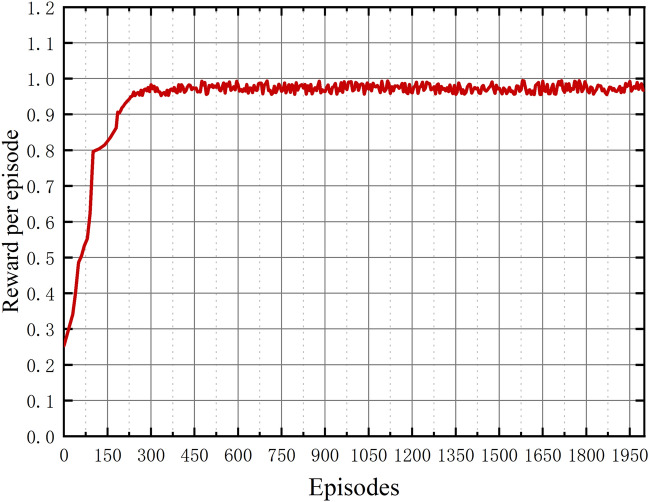
Reward with training episodes of the proposed CCSMS.

Figure 4 represents the average reward of the innovative CCSMS algorithm in this paper during the training phase. The total number of rounds is set to 2000 and the results shown in the figure are obtained by taking values every 5–10 times. Due to the gradual increase of the received rewards at the beginning of the training period, and of course the initialization of the parameters, the obtained values are not yet ideal and satisfactory, and as the training continues to intensify, the reward values become more and more stable and converge after 400 episodes, thus confirming the efficiency of the CCSMS method for solving the problem.

**Figure 5:**
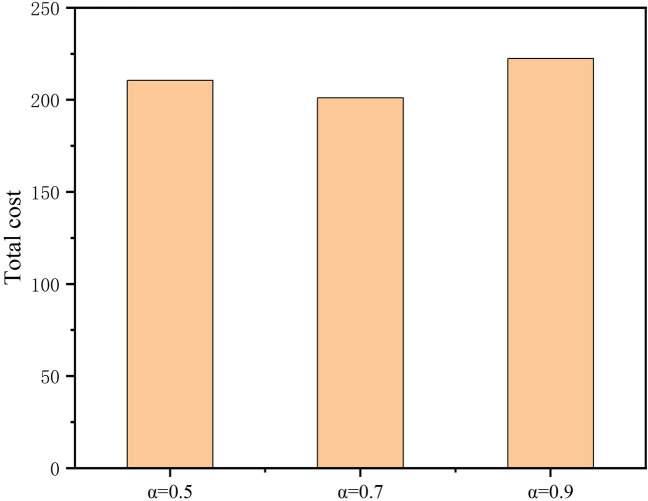
Performance on the choice of weight $\alpha$ for CCSMS.

In order to set a suitable weight factor α to weigh the relationship between delay and cost, we will next analyze the relationship between the value of α and the total overhead. As shown in Fig. 5, when α is too large or too small, it leads to poor convergence, and the best total overhead is obtained when α takes the value of 0.7. In this case, we set α = 0.7 in the following simulation experiments to try to achieve the best delay and cost overhead.

In order to set a suitable weight factor α to weigh the relationship between delay and cost, we will next analyze the relationship between the value of α and the total overhead. As shown in Fig. 5, when $\alpha$ is too large or too small, it will lead to poor convergence, and the best total overhead is obtained when $\alpha$ takes the value of 0.7. In this case, we set $\alpha =0.7$ in the following simulation experiments to try to achieve the best delay and cost overhead.

#### Analysis of task completion rate, average number of failures, and average completion time

- Average completion time: indicates the time to migrate from the original server to the target server, which is the total delay of the system;
- Task completion rate: indicates the success rate of service migration;
- Average number of failures: indicates the number of service migration failures, which, of course, is closely related to the cost overhead.

**Figure 6:**
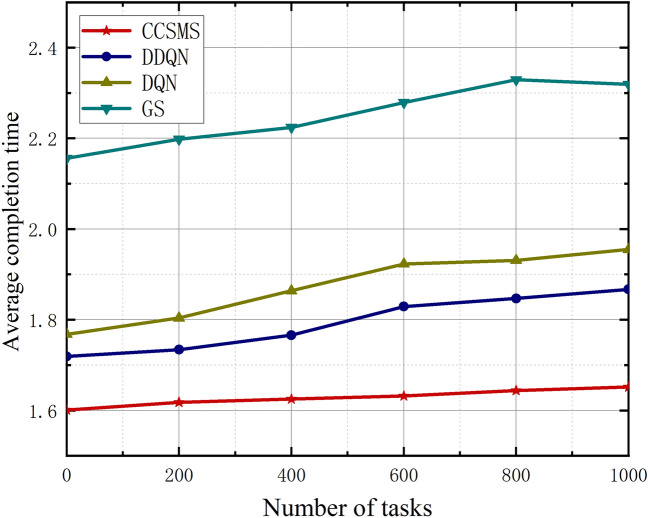
Plot of task numbers versus average completion time.

**Figure 7:**
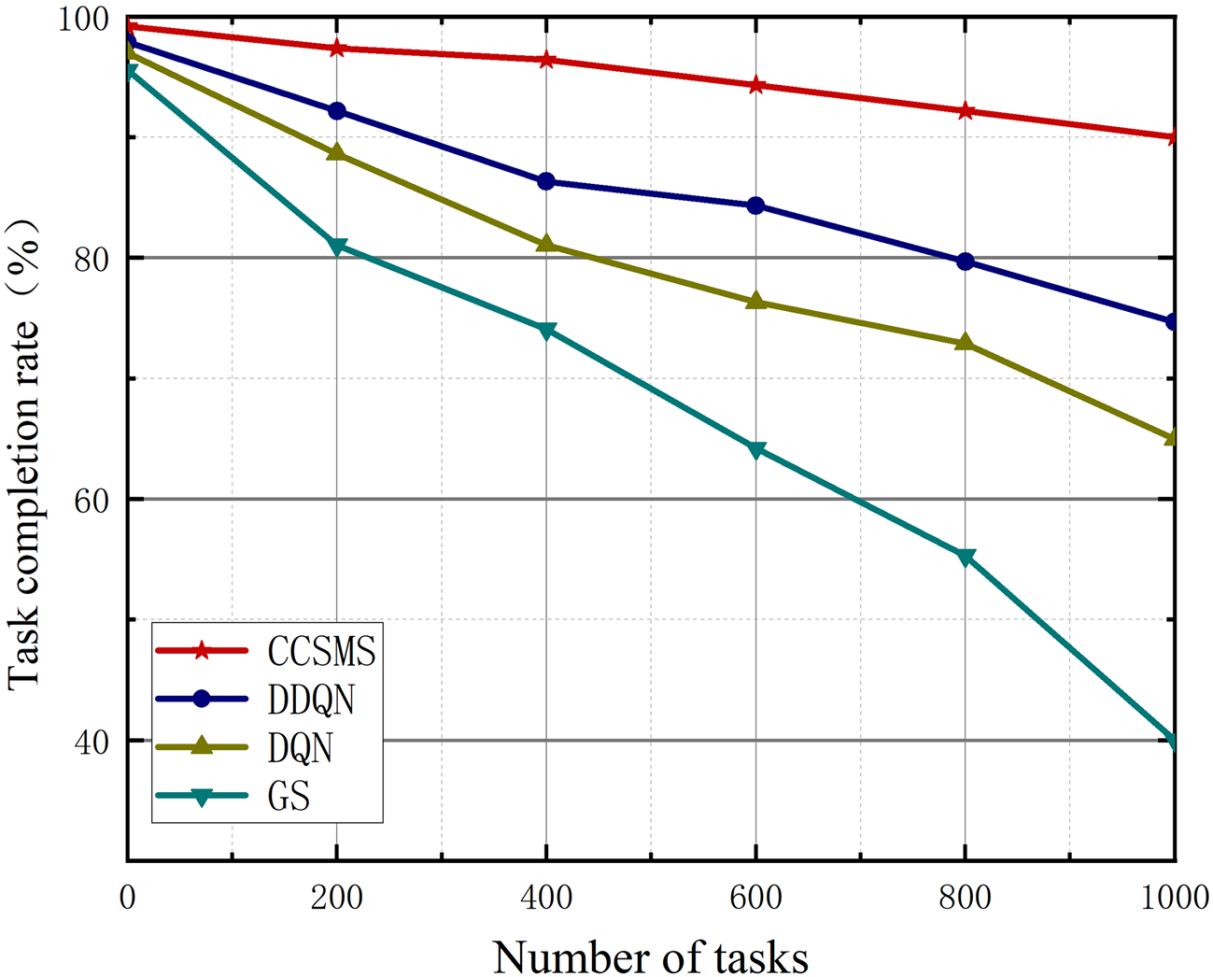
Plot of task numbers versus task completion rate.

**Figure 8:**
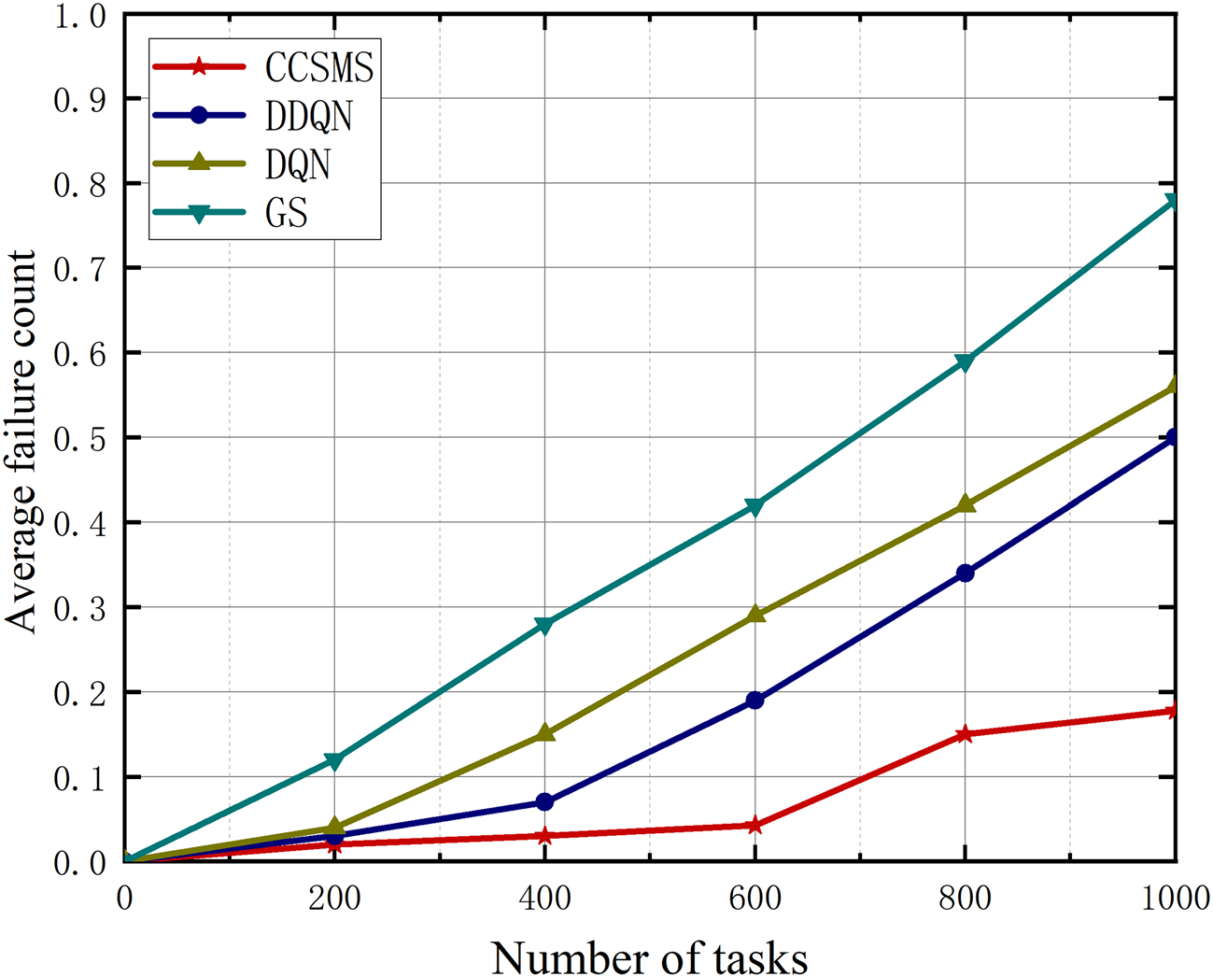
Plot of task numbers versus average failure count.

In this section, Figs. 6, 7 and 8 show us the graphs of the relationship between the average completion time, the task completion rate, and the average number of failures as the task number increases. As shown in Fig. 6, it demonstrates the curve of variation of average completion time of different algorithms with increasing task volume. The average completion delay of all the algorithms becomes an increasing trend as the task volume increases, however, the CCSMS algorithm proposed in this paper has the best performance in terms of average completion time, which is significantly less than that of DQN, DDQN and GS algorithms. Compared with the traditional methods, the algorithm significantly reduces the average completion time while the performance has a substantial improvement, which proves the effectiveness of the algorithm.

Figure 7 represents the relationship curve of task completion rate as the task volume increases. When the number of tasks is small, the completion rates of the four algorithms are almost equal, and for the increase of the number of tasks, the gap between the GS algorithm and the other three task completion rates is getting bigger and bigger. The migration algorithm adopted in this paper can effectively improve the optimization of the problem in the group motion state space environment due to the maximum entropy strategy. Compared with other methods, the strategy proposed in this paper provides more than 10% improvement in the completion rate. The CCSMS algorithm provides 15.3% improvement in the task completion rate over the DDQN algorithm, 25% over the DQN, and 49.97% over the GS when the number of tasks reaches 1000.

Figure 8 represents the curve of the average number of failures as the number of tasks increases. The proposed migration strategy provides a smaller failure rate compared to the DDQN algorithm, where the number of task failures first increases as the task volume increases, however, as the number of training sessions increases and then remains almost stable. This is due to the fact that when there is a sudden increase in the number of tasks, more tasks will be backlogged into the task queue. At the same time, in order to increase the reward and reduce the failure rate, this is when the controller will allocate more computational resources through business scheduling. With the increasing number of trainings, the service prediction for CIDS is more accurate, and the number of failures will also level off compared to the other three algorithms, which also gives our strategy a higher robustness.

#### Total delay and total overhead analysis

**Figure 9:**
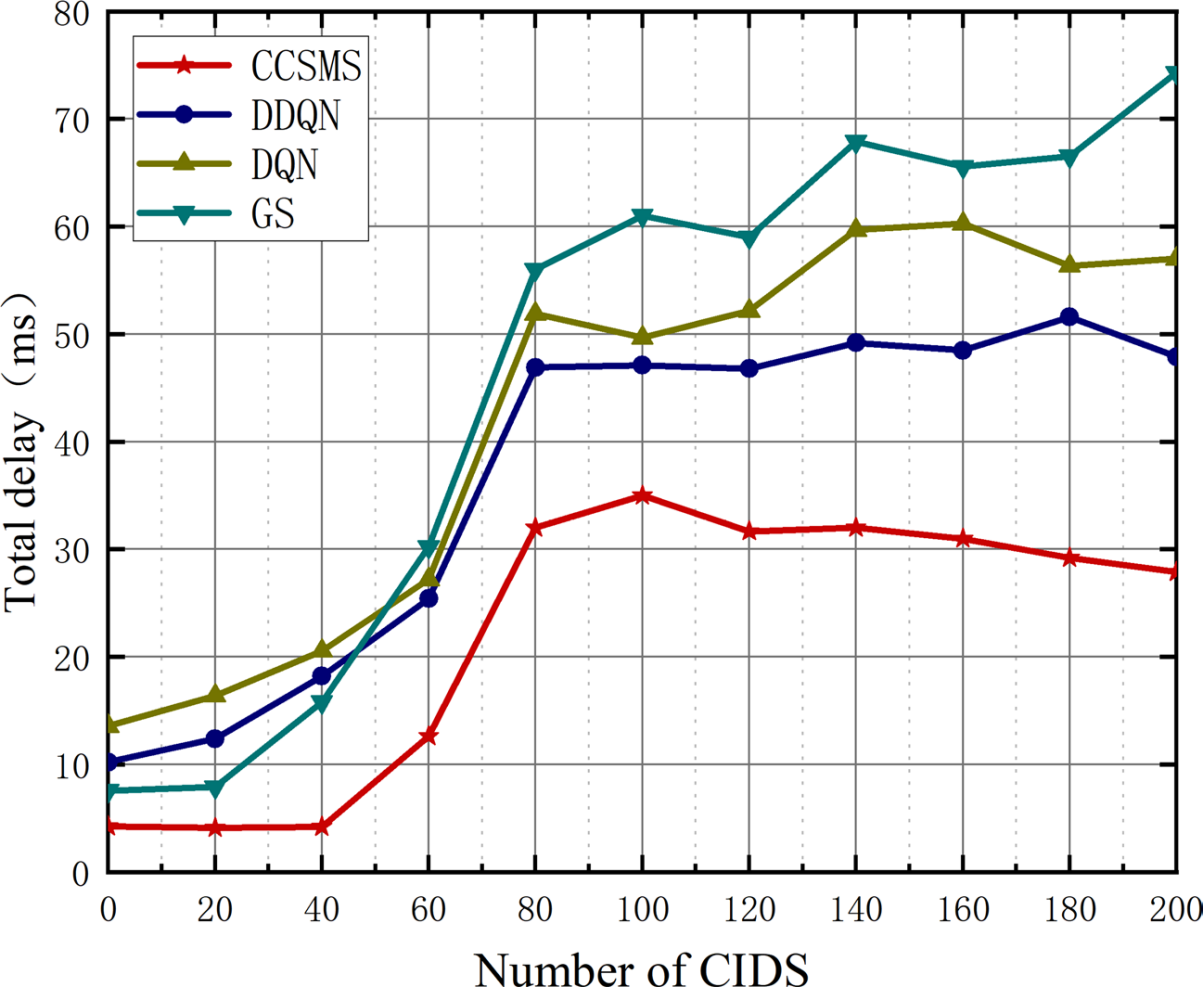
Plot of CIDS versus total delay.

Figure 9 shows the trend of the total delay of the four algorithms with the increase in the number of CIDS devices. When the number of CIDS is greater than 40, the delay increases significantly, but the CCSMS algorithm proposed in this paper still maintains a low delay. With the CIDS growth, the network will be under great pressure, and with the aggregation behavior of group wise devices, some edge servers cannot provide more services, so the algorithm will migrate the services to the less loaded areas through the controller, which increases some of the latency, resulting in a significant change in the curve.

**Figure 10:**
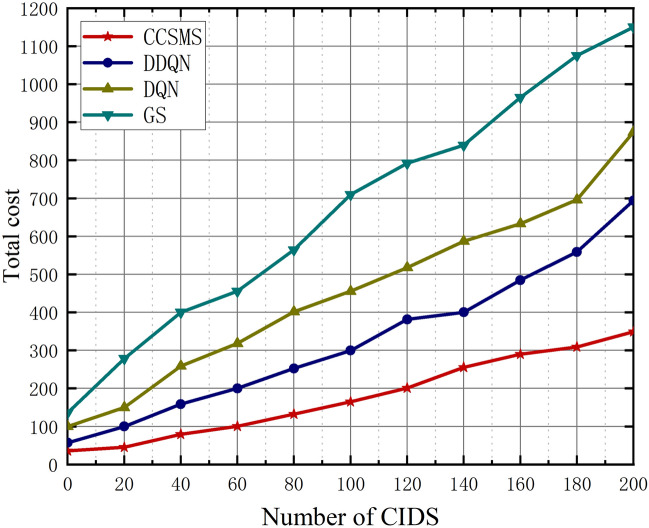
Plot of CIDS versus total cost.

We compare the total overhead of the four algorithms in Fig. 10. As the number of CIDS keeps increasing, the total cost also increases. The total cost of the CCSMS-based algorithm remains the lowest and is always lower than that of DDQN, DQN, and GS. when the number of CIDS reaches 200, the total cost of CCSMS is 24.9%, 30.1%, and 34.8% lower than that of DDQN, DQN, and GS, respectively. Our improved algorithm performs excellently in terms of cost savings because it can dynamically cluster and centralize resources for group migration, which greatly improves the efficiency of service migration.

#### Group interruption rate analysis

**Figure 11:**
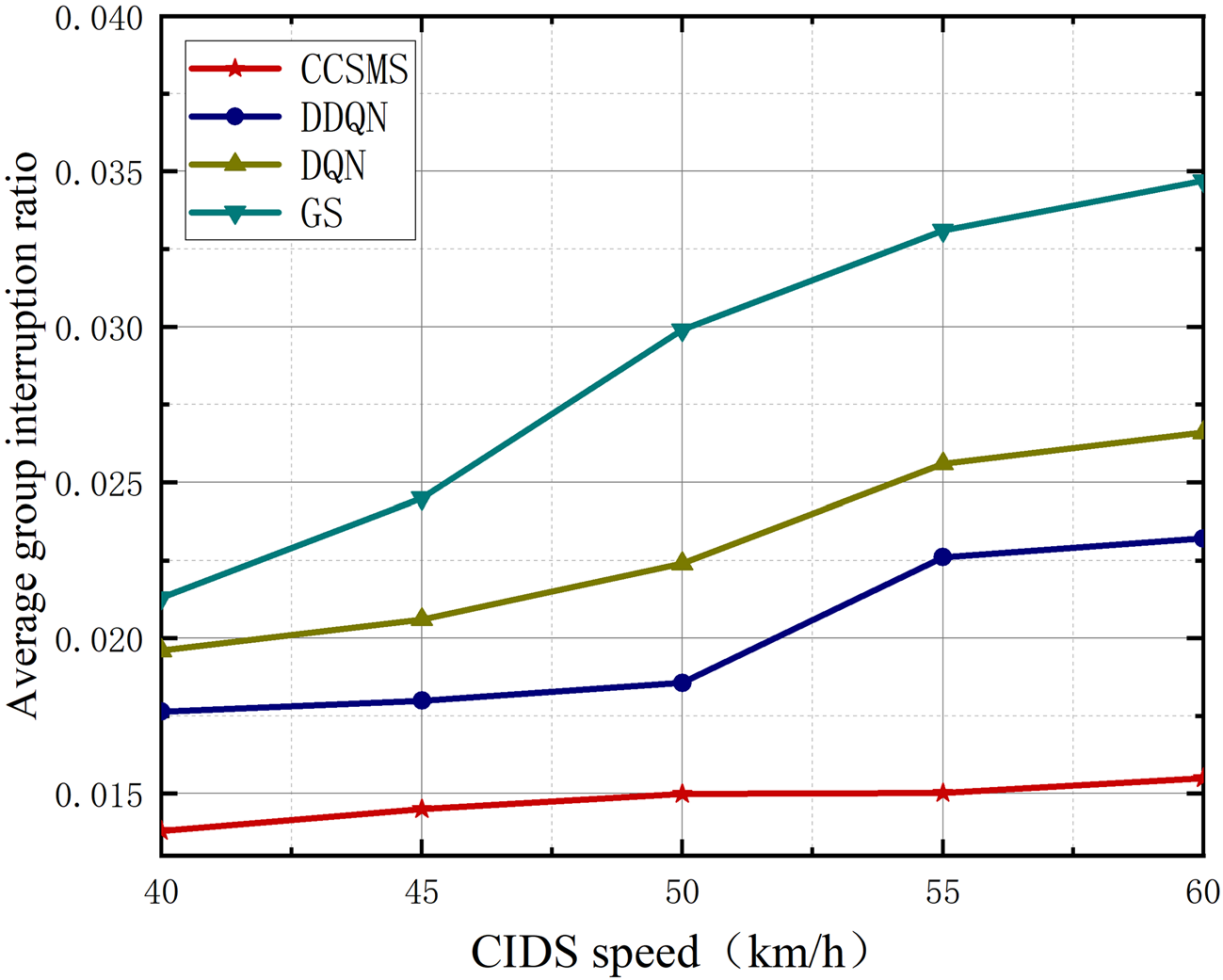
Plot of CIDS speed versus average group interruption ratio.

During the whole process of migration, after the switching command is executed, the source server stops serving to the CIDS until the new service is started after the successful service migration, so the time interruption due to the switching process is the group interruption time. The result of group interruption rate is shown in Fig. 11. From the figure we can analyze that the group interruption rate increases as the speed of the CIDS increases, but the interruption rate of the CCSMS method proposed in this paper is the lowest among them. Due to the different time period required to re-establish the connection, switching failure leads to longer interruption time, furthermore due to the fact that the algorithm in this paper is able to concentrate the cluster users of the same service migration request very well. Our proposed cluster head scheme can better serve the members of the group for service migration, which results in a very low level of termination in this scheme and greatly reduces the level of interruption in the service migration strategy.

## Conclusion

Focusing on a collaborative service migration strategy tailored for CIDS clustering in high-speed mobile mode, this paper presents an innovative approach. We introduce an innovative inter-cluster collaborative optimization approach. Modeling the problem as a partially observable Markov decision-making process, and the objective problem as a mathematical model of latency and cost overheads. To address the challenges posed by the optimization dimension catastrophe, we migrate the Soft Q-Learning algorithm to a multi-intelligence environment and propose a multi-intelligence deep reinforcement learning algorithm, called CCSMS. Leveraging the clustering model, members sharing similar migration intentions are grouped and strategically planned, which in turn optimizes resource allocation and scheduling through control collaboration, significantly reducing system latency and enhancing system convergence. Compared with other existing classical algorithms, it exhibits superior performance in terms of delay reduction, cost overhead minimization, migration rate optimization, and robustness enhancement. Looking ahead, our future research will prioritize load balancing, security, and robustness enhancements, leveraging artificial intelligence technology.

## Data Availability

The data that support the findings of this study are available from the corresponding author, [Zhi Yu], upon reasonable request.
